# Construct-related validity of the strengths and difficulties questionnaires with three and five dimensions: A multitrait-multimethod analysis

**DOI:** 10.1177/13591045231168703

**Published:** 2023-04-05

**Authors:** Francis Anne Carneiro, Pedro A Costa, Isabel Leal

**Affiliations:** William James Center for Research, 56068 ISPA – University Institute, Lisbon, Portugal

**Keywords:** Strengths and difficulties questionnaire, multitrait-multimethod, multi-informants, convergent-related validity, discriminant-related validity

## Abstract

The Strengths and Difficulties Questionnaire (SDQ) is one of the most broadly used questionnaires to evaluate children’s psychological adjustment, however its internal structure has been a target of ongoing controversy. Recent studies suggested a three-factor structure of the SDQ, however data is still scarce. The present study used the Multitrait-Multimethod analysis to examine SDQ construct related-validity with three and five dimensions, provided by children, their parents and teachers. A total of 415 participants were recruited from a Portuguese community sample. Both SDQ versions presented good convergence-related validity, with higher values for the five version. Findings from this study suggest that the SDQ with three dimensions could be more suitable as a screening measure of children’s psychological adjustment in a community low-risk sample. Nevertheless, the SDQ still needs further psychometric improvements in order to properly collect information from multi-source samples about the prevalence of children’s psychological adjustment.

The Strengths and Difficulties Questionnaire (SDQ) is one of the most broadly used brief questionnaires to evaluate psychological adjustment in children and adolescents aged between 2 and 17 years (Achenbach et al., 2008). The SDQ has been translated into more than 80 languages and can be completed by teachers and parents of children from 4 to 16 years old, and by children/adolescents from 11 to 16 years old. It is composed by 25 items that could be organized into five dimensions namely Emotional Problems, Peer Problems, Conduct Problems, Hyperactivity/Inattention and Prosocial Behaviours. Another SDQ version was further developed by Goodman and colleagues (2010) in which the SDQ could be organized into three dimensions, namely Externalizing, Internalizing and Prosocial Behaviours. In this version the Emotional Problems and Peer dimensions merge into a subscale named ‘Internalizing Problems’ and the conduct and Hyperactivity/Inattention dimensions merge into an ‘Externalizing Problems’ subscale. The two merged scales are more suitable to assess children’s psychosocial adjustment from community samples, whereas the five separate scales could provide more useful information in high-risk and/or clinical samples (Goodman et al., 2010).

Nevertheless, the SDQ internal structure has been a target of ongoing controversy. Several studies with children/adolescents, parents and teachers as independent informants have reported adequate support for the five-factor structure version (e.g. Stone et al., 2010), whilst other studies have either found only marginal support (e.g., Hill & Hughes, 2007) or no support for this SDQ version (e.g., Dickey & Blumberg, 2004). Some studies showed difficulties in confirming the SDQ five-factor structure and revealed poor construct validity (Pechorro et al., 2011). Other studies with SDQ five-factor structure and with three informants (self-report, teacher and parent) have found no evidence of SDQ discriminant-related validity (e.g. Marzocchi et al., 2004).

The three-factor SDQ structure has been supported in some exploratory analyses in the US (parents), Belgium (parents and children), and Finland (youth SDQ) (Dickey & Blumberg, 2004; Koskelainen et al., 2001; Van Leeuwen et al., 2006). In Portugal, Costa and colleagues’ (2020) study showed evidence of acceptable internal consistencies the three dimensions and evidence of discriminant-related validity, however poor convergent-related validity. This issue about the best psychometric version of SDQ should be investigated in detail.

Another enduring issue is whether different informants are truly indicating the same construct (Munkvold et al., 2009). A study with multinational sample of children aged between 6 to 11 years-old from seven European countries have found only moderate agreement between parent and teacher SDQ ratings (Cheng et al., 2018), whilst Hill and Hughes (2007) have found evidence for convergency among parent, teacher and peer ratings with first graders at risk of educational failure.

Construct validity is an important aspect of validity assessment of a measurement, and it can be evaluated through convergent- and discriminant-related validities (Byrne, 2010). Research about construct validity usually focuses on the extent to which data exhibit evidence of convergent-related validity (the extent to which different methods correspond in their assessment of the same trait), discriminant-related validity (the extent to which independent methods differ in their assessment of different traits) and method effects (an extension of the discriminant-related validity issue; Campbell & Fiske, 1959). Convergent- and discriminant-related validities can be more robustly evaluated by using the multitrait–multimethod (MTMM) method (Widaman, 1985).

To date, eight studies (Table 1) examined construct-related validity of the SDQ five-factor structure, but none assessed it for the SDQ three-factor structure with the MTMM approach. Although Goodman (et al., 2010) recommended a three-factor structure the analysis was only performed with the Internalizing and Externalizing dimensions. Regarding the SDQ five-factor structure, most of the studies found good convergent-related validity and discriminant-related validity problems for most dimensions.

**Table 1.** Summary of Studies Assessing Strengths and Difficulties Questionnaire with a Multitrait-Multimethod Approach.

Authors	SDQ version	Analysis	Sample	Main findings
Erhart et al., 2009	5 dimensions	MTMM based on the original Campbell and Fiske (1959) approach	1700 children/adolescents and parents; Germany	Lower convergent-and discriminant-related validity for the phone survey methods
Gaete et al., 2018	5 dimensions	CFA approach to MTMM analysis	1284 adolescents and parents; Chile	Five-factor model as a suitable version
	5 dimensions	MTMM minus one model analysis	202 adolescents, mothers and teachers; Australia	Good convergent-related validity; good discriminant-related validity (except between conduct problems and Hyperactivity/Inattention)
Goodman et al., 2010	5 dimensions; internalizing and externalizing dimensions	MTMM based on the original Campbell and Fiske (1959) approach	18,222 Children/Adolescents, parents and teachers; United Kingdom	Five-factor structure: Good convergent-related validity; low discriminant-related validity in some dimensions. Internalizing and externalizing dimensions: Good convergent- and discriminant-related validity
Hill & Hughes, 2007	5 dimensions	MTMM based on the original Campbell and Fiske (1959) approach	784 children, parents and teachers; United States of America	Five-factor structure: Good convergent-related validity and low discriminant-related validity in some dimensions
Smid et al., 2018	5 dimensions	MTMM based on the original Campbell and Fiske (1959) approach	8806 Children/Adolescents, parents and teachers; Norway	Evidence for convergent- and discriminant-related validity
Van Roy et al., 2008	5 dimensions	CFA approach to MTMM	26,269 children/adolescents and parents; Norway	Five-factor model as a suitable version
Yu et al., 2016	5 dimensions	CFA– MTMM model	327 parents (Chinese and Korean immigrants) and teachers. United States of America	Evidence of convergent-related validity; good discriminant-related validity (except between conduct problems and Hyperactivity/Inattention)

Despite useful findings from the aforementioned studies data is still inconsistent for the SDQ five-factor structure, and scarce to the three-factor version. The main purpose of this study is to assess construct validity for SDQ with three and five dimensions, provided by children, their parents and teachers, and within the framework of a multitrait-multimethod (MTMM) design. The specific goals are to evaluate the internal consistency, convergent- and discriminant-related validity of SDQ with three dimensions and five dimensions. The two versions of SDQ were explored since the three-dimension version could be more appropriate to evaluate psychological adjustment in low-risk community samples. Goodman (et al., 2010) also recommended the importance of using multiple approaches to assess construct validity in order to obtain a more and complete information about SDQ performance, however, until this date very few studies have explored SDQ construct-related validity with a sample of children and their respective parents and teachers simultaneously, within a MTMM design.

## Method

### Participants

This community sample comprised a total of 415 participants, including 136 children, 142 parents (96 mothers, 46 fathers) and 137 teachers. Inclusion criteria were having Portuguese nationality and having at least one child whose age was between 10 to 15 years. Children were in the 5th to 9th grade in primary school, their mean age was 12 years (*SD* = 1.709), and more than half were boys (54.4%). Parents’ ages ranged from 36 to 63 years old (*M* = 44.970; *SD* = 4.479) and the overwhelming majority was married, full-time employed, held a college degree, and lived in an urban area (for more details see Table Appendix A).

### Measures

Before completing the study measures, parents were asked to complete a brief sociodemographic questionnaire which included individual questions (age, relationship with the children, marital status, educational level, professional status, partner’s educational level, partner’s professional status and residential area) and family characteristics (child’s age and gender, child relationship with the partner and household composition).

#### Strengths and Difficulties Questionnaire (SDQ)

Children, parents, and teachers completed the self-report, parent and teacher version, respectively, of the Portuguese version of Strengths and Difficulties Questionnaire (Fleitlich et al., 2005). SDQ assesses children’s psychosocial adjustment, relationships, emotions and behaviours. The SDQ is composed by 25 items and was originally created with five scales, namely, Emotional Problems [EP], Peer Problems [PP], Conduct Problems [CP], Hyperactivity [HY], and Prosocial Behaviours [PB]. Recent studies suggested a three scales version, as being more suitable for low-risk community samples and composed by Internalizing Problems [I] (combining the EP and the PP scales), Externalizing [E] (combining the CP and HY scales) and Prosocial Behaviours [PB]. Each dimension is scored on a three-point scale (0 = Not true; 1 = Somewhat true; 2 = Certainly True) and ranging from 0 to 10, and total difficulties score ranging from 0 to 40. The reversed scores were performed for items 7, 11, 14, 21 e 25. Internal consistency of SDQ version will be reported in the results section.

### Procedures

Participants were recruited through non-probabilistic intentional sampling in private schools, post-class educational centres and soccer learning centres from the Lisbon metropolitan area. Participants completed the questionnaires via Paper and Pencil (P&P) or online, as some schools and parents requested an online version. During the sample recruitment, some schools and learning centres have only returned the parents’ questionnaires (*n* = 55) and therefore those participants were excluded from the present study, since the purpose was to match children with their respective parent and teacher. Data from parents that did not respond to the sociodemographic measures that allowed us to make the match with the children and teacher were also excluded from the study. In some cases, it was not possible to recruit the three informants (14 participants excluded), however the questionnaires from the two informants were included. The final sample was comprised of 129 triads (children and their respective parents and teachers) and 14 doubles (6 Children and their parents; 1 Child and their teacher; 7 Parents and Teachers of the same children). Sample recruitment occurred between November 2018 and September 2019. Following the Declaration of Helsinki, all participants were given the option to elucidate any questions related to study’s content and procedures. All participants have signed a consent form prior to their participation. The study was approved by *****’s Ethics Committee (approval number D/001/03/2018).

### Data analysis

Sample size for the present study has been determined according to Saris and Galhofer (2014). The authors mentioned that the sample size of a three-group design should be higher than 300 in order to obtain design efficiency, regardless the method variance. Descriptive statistics were calculated for all items of the SDQ using SPSS (v. 25, SPSS Inc. Chicago, IL). Items’ sensitivity was evaluated through Skewness (Sk) and Kurtosis (Ku) analysis. Absolut values of |Sk| and |Ku| greater than three and seven, respectively, were considered as a severe violation of the normality assumption (Marôco, 2014). SDQ internal consistency was evaluated through standard Cronbach’s alpha coefficient (Marôco, 2014).

**Figure 1.**
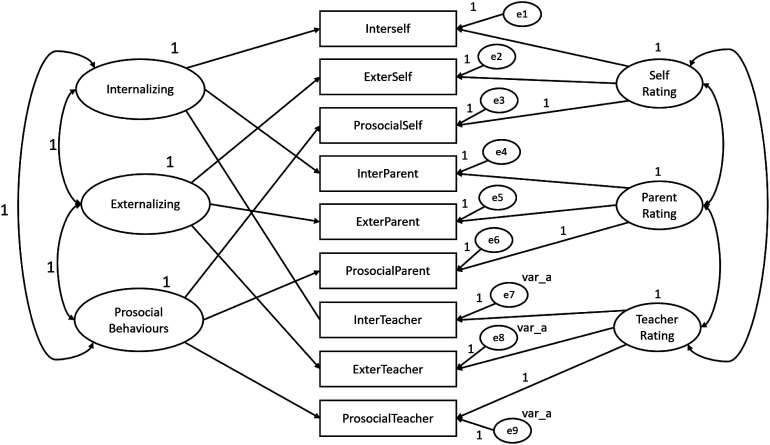
SDQ 3 dimensions hypothesized MTMM general CFA model (Model 1: freely correlated traits; freely correlated methods).

**Figure 2.**
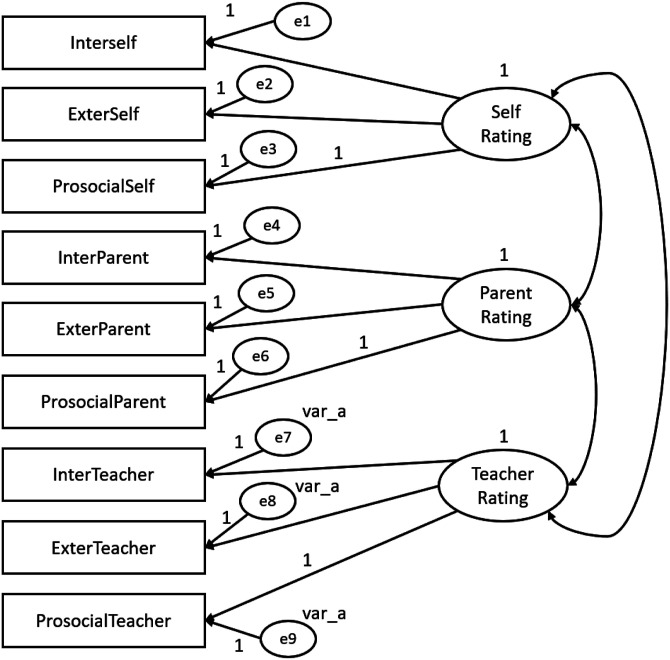
SDQ 3 dimensions MTMM Model 2 (no traits; correlated methods).

**Figure 3.**
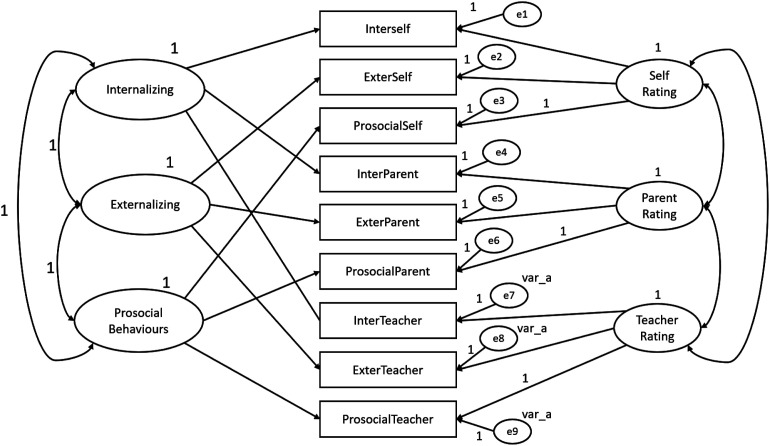
SDQ 3 dimensions MTMM model 3 (perfectly correlated traits; freely correlated methods).

**Figure 4.**
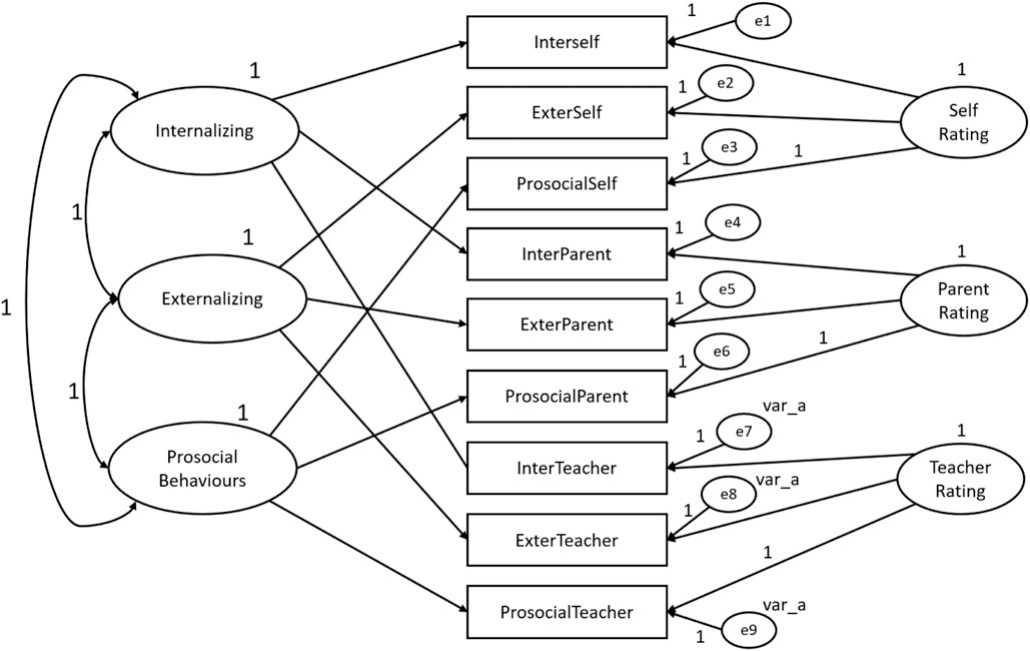
SDQ 3 dimensions MTMM model 4 (freely correlated traits; uncorrelated methods).

In order to test for evidence of SDQ construct-related validity, convergent- and discriminant-related validity were tested within the framework of a multitrait-multimethod (MTMM) design by which multiple traits (SDQ dimensions) were measured by multiple methods (children, parents and teachers). The MTMM analysis was conducted for both matrix level and individual parameter level and were performed with the SDQ constituted by three dimension and by five dimensions. The following steps were performed according do Byrne’s guidelines (2010) and all MTMM analysis were conducted using AMOS program (v. 18, SPSS Inc. Chicago, IL). Four models were created using SDQ dimensions as traits and the three informants (children, parents and teachers) as methods**.** The first model (Model 1) is represented by both trait and method factors and includes correlations between traits and correlations between methods. Model 1 also represents the hypothesized model and was the baseline against which the other nested and more restricted models were compared. Model 2 corresponds to an absence of trait factors. Model 3 includes trait and method factors, although it includes traits that are perfectly correlated. Similarly, to Model 1 and 2, in Model 3 the method factors are freely estimated. Model 4 is different from Model 1 only in the absence of specified correlations between method factors. Specification of parameters of the SDQ with three dimensions for MTMM Model 1, 2, 3 and 4 is portrayed schematically in Figures 1–4 respectively.

#### MTMM matrix-level analyses

The goodness-of-fit indices (χ2 and CFI) of Model 1 were compared with the goodness-of-fit indices of the other MTMM Models to evaluate the existence of evidence of convergent- and discriminant-related validity on a matrix level. To determine convergent-related validity, or the extent that independent measures of the same trait are correlated (e.g., parent rated and self-rated prosocial behaviours), comparisons between Model 1 and Model 2 were performed using the difference in CFI and χ^2^ values. A significant difference in χ^2^ (Δχ^2^) and in CFI (ΔCFI) and a ΔCFI greater to .01 provides evidence of convergent-related validity (Byrne, 2010). Discriminant-related validity was evaluated for traits and methods. To examine traits’ discriminant-related validity (the extent to which *independent measures* of *different traits* are correlated), Model 1 and Model 3 were compared*.* A large Δχ2 and/or a substantial ΔCFI provides support for discriminant-related validity. To examine methods’ discriminant-related validity, Model 1 and Model 4 were compared. A small Δχ2 and/or a small ΔCFI indicates discriminant-related validity.

#### MTMM Parameter-level analyses

A more precise evaluation of trait- and method-related variance can be determined by examining individual parameter estimates of the factor loadings and factor correlations of the hypothesized model (Model 1). Convergent-related validity was assessed through the factor loadings. The magnitude of the trait loadings reflects the convergent-related validity and an overall comparison of trait and method loadings reveals the proportion of method variance that may exceed the trait variance. If this proportion is significant, convergent-related validity could be weakened. Discriminant-related validity bearing on specific traits and methods is determined by examining the factor correlation matrices. In order to suggest discriminant-related validity, the correlations between traits should be negligible. Regarding method’s factor correlations, their discriminability is related to the extent to which they are maximally dissimilar (correlations should be also negligible).

The mentioned analysis will be performed with SDQ with three dimensions and SDQ with five dimensions.

## Results

### Missing data

There were 84 scores randomly missing from different participants within a total of 8715 scores. The missing value were approximately 1%. Expectation maximization (EM) was used to impute the missing data.

### Descriptive statistics of strengths and difficulties questionnaire individual items

Children descriptive statistics for the SDQ items indicated that the three-point Likert scale, with answers ranging from zero to two, was used for almost all items, except for items 12 (“I *fight a lot*”) and 17 (“I am kind to younger children”). The answers “certainly true” and “Not true” were not used for any children in items 12 and 17, respectively. Skewness (.095< |Sk|< 2.762) and Kurtosis (.343 < |K| < 6.696) values did not show evidence of violations of the normal distribution (Kline, 2016). Average score for SDQ items ranged from .10 (*SD* = .305) and to 1.83 (*SD* = .376). Parents descriptive statistics for SDQ items indicated that the three-point Likert scale was used for almost all items, except for item 1 (“Considerate of other people’s feelings”). The answer “Not true” was not used for any parent to respond to item 1. Parents SDQ item’s distribution did not present acceptable skewness and Kurtosis for items 12 (|Sk| = 6.820; |K| = 49.904), 17 (|Sk| = 4.223; |K| = 18.867), and 22 (|Sk| = 6.765; |K| = 49.319). The other items presented acceptable skewness (.018< |Sk|< 2.470) and kurtosis (.042 < |K| < 5.522) values (Kline, 2016). The average score for SDQ items ranged from .04 (*SD* = .222) and to 1.93 (*SD* = .285). Teachers’ descriptive statistics for SDQ items indicated that the three-point Likert scale was used for all items. Teachers’ SDQ items distribution did not present acceptable skewness and Kurtosis for items 11 (“Has at least one good friend”) (|Sk| = 3.298; |K| = 49.904), and 22 (“Steals from home, school or elsewhere”) (|Sk| = 3.813; |K| = 14.854). The other items presented acceptable skewness (.296< |Sk|< 2.208) and Kurtosis (.026 < |K| < 4.187) values (Kline, 2016). The average score for SDQ items ranged from .09 (*SD* = .340) and to 1.71 (*SD* = .513).

### Strengths and difficulties questionnaire with three dimensions

#### Internal consistency

Teacher’s ratings presented the highest internal consistencies, especially for E and PB (α_Internalizing_ = .798; α_Externalizing_ = .857; α_Prosocial_ = .803). Parents’ ratings presented low internal consistencies for all dimensions (α_Internalizing_ = .732; α_Externalizing_ = .773; α_Prosocial_ = .653). Children’ ratings presented the lowest internal consistencies with low values for I and E (α_Internalizing_ = .675; α_Externalizing_ = .694), and very low (unacceptable) internal consistency for PB (α_Prosocial_ = .579).

#### Convergent and discriminant-related validity: MTMM matrix-level analyses

Table 2 presents the summary of goodness of fit indices of Model 1, Model 2, Model 3 and Model 4. Model 1 showed a very good fit to the data while for Model 2 goodness-of-fit is very poor. Model 3 presented a marginally good fit but not as well-fitted as Model 1. Model 4 revealed good fit to the data but slightly less well-fitted than Model 1.

**Table 2.** Summary of Goodness-of-Fit Indices for SDQ MTMM Models.

Model	RMSEA					
	χ^2^	Df	CFI	RMSEA	90%C.I.	*p*
1. Freely correlated traits; ^a^ freely correlated methods	22.800	17	.976	.05	.000, .093	.492
2. No traits; freely correlated methods	118.582	29	.628	.143	.116, .170	.000
3. Perfectly correlated traits; freely correlated methods	50.108	20	.875	.100	.065, .134	.011
4. Freely correlated traits; uncorrelated methods	31.596	20	.952	.062	.006, .101	.291

^a^Respecified model with an equality constraint imposed between E7, E8 and E9.

In order to evaluate convergent- and discriminant-related validity at a matrix level, a summary of comparisons between Model 1 with Models 2, 3 and 4 were performed. The Δχ^2^ is highly significant (χ^2^_(12)_ = 95.782, *p* < .001), and the difference in practical fit (ΔCFI = .348) is significantly above .01, which suggested strong evidence of convergent-related validity. The comparison between Model 1 and Model 3 yields a Δχ^2^ value that is statistically significant (χ^2^_(3)_ = 27.308, *p* < .001) and the difference in practical fit was fairly large (ΔCFI = .101), suggesting modest evidence of discriminant-related validity for SDQ traits. The comparison between Model 1 and Model 4 yield a Δχ^2^ value that was small and statistically significant (χ^2^_(3)_ = 31.59, *p* < .001) and the difference in practical fit was also small (ΔCFI = .024), which indicates evidence of good discriminant-related validity for the methods.

#### Convergent and discriminant-related validity: MTMM parameter-level analyses

As indicated in Table 3, almost all the traits’ loadings were statistically significant, except for children’s self-ratings of PB and parent ratings of PB. Teacher ratings of E presented the highest magnitude of trait loadings. Self-ratings of E presented the lowest magnitude within the statistically significant traits loading. The comparison between traits’ factor loadings and methods’ factor loading, revealed that the proportion of method variance exceeds the trait variance for two of the self-ratings (E and PB), for all parent ratings and one of the teacher ratings (PB). The attenuation of traits by methods (mostly associated to parent ratings) showed a moderate convergent-related validity.

**Table 3.** Trait and Method Loadings for MTMM Model 1 (Correlated Traits; Correlated Methods).^a^

	I	E	PB	SR	PR	TR
	**Self-ratings (SR)**					
Internalizing (I)	.461			−.435		
Externalizing (E)		.470		−.521		
Prosocial behaviours (PB)			.027^b^	−.696		
	**Parent ratings (PR)**					
Internalizing (I)	.474				−.488	
Externalizing (E)		.480			−.592	
Prosocial behaviours (PB)			.150^c^		.643	
	**Teacher ratings (TR)**					
Internalizing (I)	.697					−.583
Externalizing (E)		.918				−.232^d^
Prosocial behaviours (PB)			.494			.506

^a^Standardized estimates.

^b^Not statistically significant (*p* = .826).

^c^Not statistically significant (p = . 218).

^d^Not statistically significant (*p* = .144).

As shown in Table 4, the traits were significantly associated, except for I with PB. I and E presented a significant and low association, whilst the E and PB presented a moderate and negative association. Finally, method factor correlations presented significant correlations. The self-ratings with both parent and teacher ratings presented low associations, however the association between teacher and parent ratings was moderate. Those correlations indicated a modest level of discriminant-related validity of the methods.

**Table 4.** Trait and Method Correlations for MTMM Model 1 (Correlated Traits; correlated Methods).^a^

	Traits			Methods		
Measures	I	E	PB	SR	PR	TR
Internalizing (I)	1.000					
Externalizing (E)	.463	1.000				
Prosocial behaviours (PB)	.054^b^	−.662	1.000			
Self-Ratings (SR)				1.000		
Parent Ratings (PR)				.368	1.000	
TeacherRatings (TR)				.399	.615	1.000

^a^Standardized estimates.

^b^Not statistically significant (*p* = .847).

### Strengths and difficulties questionnaire with five dimensions

#### Internal consistency

Teacher’s ratings presented the highest internal consistencies, especially for the HY and PB (α_Emotional_ = .735; α_Conduct_ = .651; α_Hyperactivity_ = .865; α_Peer_ = .684; α_Prosocial_ = .803). Parents’ ratings presented good internal consistency for HY (α_Hyperactivity_ = .770), low internal consistency for EP, PP and PB (α_Emotional_ = .646; α_Peer_ = .635; α_Prosocial_ = .653), and very low internal consistency for CP (α_Conduct_ = .545). Children’ ratings presented the lowest internal consistency, with unacceptable values of internal consistency for CP, very low values for EP, PB and PP (α_Emotional_ = .574; α_Conduct_ = .452; α_Peer_ = .643; α_Prosocial_ = .579). HY presented low values of internal consistency (α_Hyperactivity_ = .706).

#### Convergent and discriminant-related validity: MTMM matrix-level analyses

Table 5 presents the summary of goodness of fitness indices of Model 1, Model 2, Model 3 and Model 4. For Model 1 there was a good fit to the data, while for Model 2 the Goodness-of-fit was not adequate. Regarding Model 3, although goodness-of-fit for this model is better than for Model 2, the model is marginally well-fitted and less well-fitted than Model 1. Goodness-of-fit results for model 4 revealed a good fit to the data, but slightly less well-fitted than Model 1.

**Table 5.** Summary of Goodness-of-Fit Indices for SDQ.

Model	RMSEA					
	χ^2^	Df	CFI	RMSEA	90%C.I.	*P*
1. Freely correlated; ^a^ freely correlated methods	72.779	65	.986	.029	.000 .060	.849
2. No traits; freely correlated methods	242.076	90	.717	.109	.093, .126	.000
3. Perfectly correlated traits; freely correlated methods	141.142	75	.877	.079	.059, .099	.012
4. Freely correlated traits; uncorrelated methods	83.753	68	.971	.040	.000, .067	.696

Regarding convergent- and discriminant-related validity, comparisons between Model 1 with Models 2, 3 and 4 were performed. For Model 1 and Model 2 the Δχ2 was highly significant (χ^2^(25) = 169.297, *p* < .001), and the difference in practical fit (ΔCFI = .269) was significantly above .01, which suggests strong evidence of convergent-related validity. For Model 1 and Model 3 the comparison yields a Δχ^2^ value that is statistically significant (χ^2^_(10)_ = 68.363, *p* < .001) and the difference in practical fit was large (ΔCFI = .109), suggesting modest evidence of discriminant-related validity for the traits. For Model 1 with Model 4 the comparison yields a small and significant Δχ^2^ value (χ^2^_(3)_ = 10.974, *p* = .010) and also a small difference in practical fit (ΔCFI = 0.015) which argues for evidence of good discriminant-related validity for the methods.

#### Convergent and discriminant-related validity: MTMM parameter-level analyses

All trait loadings were statistically significant with magnitudes ranging from .286 (parent-ratings of PB) to .870 (teacher ratings of PP). The comparison between traits’ factor loadings and methods’ factor loading, revealed that the proportion of method variance exceeds the trait variance for one of the self-ratings (Pb), two of the parent ratings (CP and PB) and one of the teacher ratings (PB). The proportion of methods with higher loadings than the traits correspond to 4 in 15 methods. The slight attenuation of traits by methods (mostly associated to parent ratings) and the significant magnitudes of the traits’ loadings showed a good convergent-related validity (Table 6).

**Table 6.** Trait and Method Loadings for SDQ 5 dimensions MTMM Model 1 (Correlated Traits; Correlated Methods).^a^

	EP	CP	H	PP	PB	SR	PR	TR
			**Self-ratings (SR)**					
Emotional problems (EP)	.417					−.292		
Conduct problems (CP)		.597				−.337		
Hyperactivity (H)			.731			−.162^c^		
Peer problems (PP)				.523		−.122^b^		
Prosocial behaviours (PB)					.367	.715		
			**Parent ratings (PR)**					
Emotional problems (EP)	.466						−.420	
Conduct problems (CP)		.385					−.725	
Hyperactivity (HY)			.555				−.486	
Peer problems (PP)				.551			−.381	
Prosocial behaviours (PB)					.286		.620	
			**Teacher ratings (TR)**					
Emotional problems (EP)	.572							−.523
Conduct problems (CP)		.665						−.508
Hyperactivity (HY)			.597					−.578
Peer problems (PP)				.870				−.269
Prosocial behaviours (PB)					.371			.502

^a^Standardized estimates.

^b^Not statistically significant (*p* = 2.49).

^c^Not statistically significant (*p* = .171).

As shown in Table 7, most of the traits were significantly associated, except the CP with EP, and HY with PB. PP presented moderate to high associations with CP and EP, respectively. PB presented moderate to high and negative associations with PP and CP, respectively, and HY presented high associations with CP. The mentioned associations decreased the attainment of trait discriminant-related validity. Finally, regarding the method factor correlations, the association between parent ratings and teachers’ ratings was moderate which diminished the discriminant-related validity of the methods. Nevertheless, discriminant-related validity was higher for methods that for the traits.

**Table 7.** Trait and Method Correlations for SDQ 5 dimensions MTMM Model 1 (Correlated Traits; correlated Methods).^a^

	Traits						Methods		
Measures	EP	CP		HY	PP	PB	SR	PR	TR
Emotional problems (EP)	1.000								
Conduct problems (CP)	.235^b^	1.000							
Hyperactivity (HY)	.361	.753		1.000					
Peer problems (PP)	.802	.656		.420	1.000				
Prosocial behaviours (PB)	.237^c^	−.834		−.273^d^	−.676	1.000			
Self-Ratings (SR)							1.000		
Parent Ratings (PR)							.086^e^	1.000	
TeacherRatings (TR)							−.067^f^	.493	1.000

^a^Standardized estimates.

^b^Not statistically significant (*p* = 1.59).

^c^Not statistically; significant (*p* = .452).

^d^Not statistically significant (*p* = .186).

^e^Not statistically significant (*p* = .572).

^f^Not statistically significant (*p* = .732).

## Discussion

The present study examined construct-related validity of the three and five dimensions of the SDQ in a sample of children (10–15 years), parents and teachers, within the framework of a multitrait-multimethod (MTMM) design. The first goal was to explore evidence for internal consistency, convergent- and discriminant-related validity of SDQ with three dimensions. Regarding the SDQ dimension’s internal consistency the main concern was with the low to very low self-ratings. Findings suggested that for this sample the SDQ is more reliable for the parents and teachers, rather than the children. Similarly, an Italian study (Di Riso et al., 2010) with children (8–10 years) reported unacceptable to good reliability values for the SDQ with three dimensions on the self-reports. Probably for other age range this could occur differently. The convergent- and discriminant-related validity were moderate mainly due to parents’ and children’ ratings on prosocial behaviours. Prosocial behaviours also seemed to interfere with discriminant-related validity, especially for parents’ and teachers’ ratings. Issues with this dimension were already reported in other studies (e.g. Palmieri & Smith, 2007) and were expected by Goodman et al., (2010). The items of prosocial behaviours are rated in another format response and are interspersed with the items of the other dimensions (e.g. Achenbach et al., 2008).

Another goal was to explore evidence for internal consistency, convergent- and discriminant-related validity of SDQ with five dimensions. Internal consistency values were unacceptable for parents’ ratings and self-ratings. For teachers’ ratings the values were very good, except for the conduct problems dimension. Since the children of this study belong to a community sample problematic behaviours could not exist, or certain problems could not be identified because they could not have the enhanced self-reflection about their own behaviours. Teachers probably could have more privileged access to children’s behaviours in their multiple life areas comparatively to parents that usually observe them in their household and family moments. Therefore, the SDQ could be faithfully reflecting different children’s behaviours observed in different contexts from different informants’ perspectives.

The findings revealed a good convergent-related validity and low discriminant-related validity. Similarly with (Gomez, 2014), conduct problems and hyperactivity/inattention contributed to the decreasing of the discriminant-related validity. Emotional and peers' problems also contributed to this decreasing suggesting that these dimensions are confounded with each other possibly because is not expected for these children to manifest disruptive behaviours (lying or stealing) nor clinical emotional problems.

In sum, although the reliability of the SDQ with three dimensions in not satisfactory for all informants the values of internal consistency were more suitable for this sample when compared to the SDQ with five dimensions since it presented unacceptable values in self-ratings and parents’ ratings. Also, when SDQ traits are combined into higher order dimensions it helps improve discriminant-related validity indicating that the SDQ with three dimensions could be more appropriate to evaluate psychological adjustment in non-clinical samples. Goodman et al. (2010) also found higher values of convergent and discriminant-related validities for internalizing and externalizing problems, than for the five-structure SDQ version. These dimensions have the advantage to reduce measurement error since they have a greater number of items. These findings seem to corroborate the literature that refers that the three-factor structure SDQ could be more suitable for community samples with minimal risk problematics and also more appropriate as explanatory or outcome variables in epidemiological studies (Costa et al., 2020; Goodman et al., 2010). Nevertheless, studies should further investigate whether these internal consistencies are due to cultural differences in parental and youth perceptions about the latent constructs. With the appropriate psychometrical modifications, SDQ with three dimensions could be used to collect information from multi-source samples about the prevalence of children’s psychological adjustment and with the purpose of examine the need for prevention and intervention programs (Hill and Hughes, 2007).

Results should be considered in light of study limitations. The recruitment procedures and families’ characteristics could influence the findings from this study, since it is not possible do determine the representativeness of our sample regarding the Portuguese population. Future studies should aim to recruit more diverse samples. Also, future studies should explore SDQ psychometric qualities in both clinical and community low-risk sample to confirm if SDQ is indicated to attribute clinical diagnostics and/or to be a screening instrument. SDQ external criterion should be posteriorly accessed. As far as we know, this was the first study to assess simultaneously both SDQ versions regarding their construct-related validity through an MTMM design and to explore Goodman and colleague’s hypothesis about the SDQ most suitable version for low-risk samples.

## Appendices

**Appendix A.** Characteristics of the Caregiver’s Sample (*n* = 142).

	Frequency	%
Relationship Status		
Married	107	75.4
In a relationship	4	2.8
Single	9	6.3
Divorced	18	12.7
Widowed	1	.7
Educational level		
High school or less	40	28.1
At least a college degree	99	69.7
Partner’s educational level		
No partner	22	15.5
High school or less	35	24.6
At least a college degree	76	53.5
Professional status		
Full-time employment	120	84.5
Part-time employment	2	1.4
Freelance/Independent work	6	4.2
Unemployment	6	4.2
Retirement	1	.7
Partner’s professional status		
No partner	22	15.5
Full-time	101	71.1
Part-time	5	3.5
Unemployment	5	3.5
Residential area		
Urban/big city	81	57.0
Urban/suburbs of a big city	46	32.4
Semi-urban/small city	3	2.1
Rural	7	4.9
Number of people in the household		
1	5	3.5
2	8	5.6
3	41	28.9
4	59	41.5
5	17	12.0
6	4	2.8
7	2	1.4

## References

[bibr1-13591045231168703] Achenbach T. M. Becker A. Döpfner M. Heiervang E. Roessner V. Steinhausen H. C. Rothenberger A. (2008). Multicultural assessment of child and adolescent psychopathology with ASEBA and SDQ instruments: Research findings, applications, and future directions. Journal of Child Psychology and Psychiatry, 49(3), 251–275. 10.1111/j.1469-7610.2007.01867.x 18333930

[bibr2-13591045231168703] Byrne B. M. (2010). Structural equation modeling with AMOS: Basic concepts, applications, and programming (2nd ed.). Routledge.

[bibr3-13591045231168703] Campbell D. T. Fiske D. W. (1959). Convergent and discriminant validation by the multitrait-multimethod matrix. Psychological Bulletin, 56(2), 81–105. 10.1037/h0046016 13634291

[bibr4-13591045231168703] Cheng S. Keyes K. M. Bitfoi A. Carta M. G. Koç C. Goelitz D. Otten R. Lesinskiene S. Mihova Z. Pez O. Kovess-Masfety V. (2018). Understanding parent–teacher agreement of the Strengths and Difficulties Questionnaire (SDQ): Comparison across seven European countries. International Journal of Methods in Psychiatric Research, 27(1), Article e1589. 10.1002/mpr.1589 29024371 PMC5937526

[bibr5-13591045231168703] Costa P. A. Tasker F. Ramos C. Leal I. (2020). Psychometric properties of the parent’s versions of the SDQ and the PANAS-X in a community sample of Portuguese parents. Clinical Child Psychology and Psychiatry, 25(2), 520–532. 10.1177/1359104519891759 31793797

[bibr6-13591045231168703] Dickey W. C. Blumberg S. J. (2004). Revisiting the factor structure of the strengths and difficulties questionnaire: United States, 2001. Journal of the American Academy of Child & Adolescent Psychiatry, 43(9), 1159–1167. 10.1097/01.chi.0000132808.36708.a9 15322420

[bibr7-13591045231168703] Di Riso D. Salcuni S. Chessa D. Raudino A. Lis A. Altoè G. (2010). The Strengths and Difficulties Questionnaire (SDQ). Early evidence of its reliability and validity in a community sample of Italian children. Personality and Individual Differences, 49(6), 570–575. 10.1016/j.paid.2010.05.005

[bibr8-13591045231168703] Erhart M. Wetzel R. M. Krügel A. Ravens-Sieberer U. (2009). Effects of phone versus mail survey methods on the measurement of health-related quality of life and emotional and behavioural problems in adolescents. BMC Public Health, 9(1), Article 491. 10.1186/1471-2458-9-491 20042099 PMC2809066

[bibr9-13591045231168703] Fleitlich B. Loureiro M. Fonseca A. Gaspar F. (2005). Questionário de capacidades e dificuldades (SDQ-Por). [Strengths and Difficulties Questionnaire, Portuguese version] http://www.sdqinfo.org

[bibr10-13591045231168703] Gaete J. Montero-Marin J. Valenzuela D. Rojas-Barahona C. A. Olivares E. Araya R. (2018). Mental health among children and adolescents: Construct validity, reliability, and parent-adolescent agreement on the 'Strengths and Difficulties Questionnaire' in Chile. PLOS ONE, 13(2), Article e0191809. 10.1371/journal.pone.0191809 29401472 PMC5798763

[bibr11-13591045231168703] Gomez R. (2014). Correlated trait–correlated method minus one analysis of the convergent and discriminant validities of the Strengths and Difficulties Questionnaire. Assessment, 21(3), 372–382. 10.1177/1073191112457588 22936782

[bibr12-13591045231168703] Goodman A. Lamping D. L. Ploubidis G. B. (2010). When to use broader internalising and externalising subscales instead of the hypothesised five subscales on the strengths and difficulties questionnaire (SDQ): Data from British parents, teachers and children. Journal of Abnormal Child Psychology, 38(8), 1179–1191. 10.1007/s10802-010-9434-x 20623175

[bibr13-13591045231168703] Goodman R. Ford T. Simmons H. Gatward R. Meltzer H. (2000). Using the Strengths and Difficulties Questionnaire (SDQ) to screen for child psychiatric disorders in a community sample. The British Journal of Psychiatry, 177(6), 534–539. 10.1192/bjp.177.6.534 11102329

[bibr14-13591045231168703] Hill C. R. Hughes J. N. (2007). An examination of the convergent and discriminant validity of the strengths and difficulties questionnaire. School Psychology Quarterly, 22(3), 380–406. 10.1037/1045-3830.22.3.380 18843384 PMC2562744

[bibr28-13591045231168703] Kline R.B. (2016). Principles and practice of structural equation modeling (4th ed.). New York: The Guilford Press;.

[bibr26-13591045231168703] Koskelainen M. Sourander A. Vauras M. (2001). Self-reported strengths and difficulties in a community sample of Finnish adolescents. European Child & Adolescent Psychiatry, 10, 180–185. 10.1007/s007870170024 11596818

[bibr15-13591045231168703] Marôco J. (2014). Análise de equações estruturais: Fundamentos teóricos, software & aplicações. : Pêro Pinheiro. Report Number.

[bibr16-13591045231168703] Marzocchi G. M. Capron C. Di Pietro M. Duran Tauleria E. Duyme M. Frigerio A. Gaspar M. F. Hamilton H. Pithon G. Simões A. Thérond C. (2004). The use of the strengths and difficulties questionnaire (SDQ) in Southern European countries. European Child and Adolescent Psychiatry, 13(Suppl 2), 40–46. 10.1007/s00787-004-2007-1 15243785

[bibr17-13591045231168703] Munkvold L. Lundervold A. Lie S. A. Manger T. (2009). Should there be separate parent and teacher‐based categories of ODD? Evidence from a general population. Journal of Child Psychology and Psychiatry, 50(10), 1264–1272. 10.1111/j.1469-7610.2009.02091.x 19490306

[bibr18-13591045231168703] Palmieri P. A. Smith G. C. (2007). Examining the structural validity of the Strengths and Difficulties Questionnaire (SDQ) in a U.S. sample of custodial grandmothers. Psychological Assessment, 19(2), 189–198. 10.1037/1040-3590.19.2.189 17563200 PMC1997309

[bibr19-13591045231168703] Pechorro P. Poiares C. Vieira R. (2011). Propriedades psicométricas do Questionário de Capacidades e de Dificuldades na versão portuguesa de auto-resposta. Revista de Psiquiatria Consiliar e de Ligação, 16/19(1/2), 99–109. https://revista.psiquiatria-cl.org/index.php/rpcl/article/viewFile/110/17#page=101

[bibr20-13591045231168703] Saris W. E. Gallhofer I. N. (2014). Design, evaluation, and analysis of questionnaires for survey research (2nd ed.). John Wiley & Sons. 10.1002/9780470165195

[bibr21-13591045231168703] Smid S. C. Hox J. J. Heiervang E. R. Stormark K. M. Hysing M. Bøe T. (2018). Measurement equivalence and convergent validity of a mental health rating scale. Assessment. Advance online publication. 10.1177/1073191118803159 PMC7545650 30288985

[bibr22-13591045231168703] Stone L. L. Otten R. Engels R. C. Vermulst A. A. Janssens J. M. (2010). Psychometric properties of the parent and teacher versions of the strengths and difficulties questionnaire for 4- to 12-year-olds: A review. Clinical Child and Family Psychology Review, 13(3), 254–274. 10.1007/s10567-010-0071-2 20589428 PMC2919684

[bibr27-13591045231168703] Van Leeuwen K. Meerschaert T. Bosmans G. De Medts L. Braet C. (2006). The Strengths and Difficulties Questionnaire in a community sample of young children in Flanders. European Journal of Psychological Assessment, 22(3), 189–197. 10.1027/1015-5759.22.3.189

[bibr23-13591045231168703] Van Roy B. Veenstra M. Clench‐Aas J. (2008). Construct validity of the five‐factor Strengths and Difficulties Questionnaire (SDQ) in pre‐, early, and late adolescence. Journal of Child Psychology and Psychiatry, 49(12), 1304–1312. 10.1111/j.1469-7610.2008.01942.x 19120709

[bibr24-13591045231168703] Widaman K. F. (1985). Hierarchically tested covariance structure models for multitrait- multimethod data. Applied Psychological Measurement, 9(1), 1–26. 10.1177/014662168500900101

[bibr25-13591045231168703] Yu J. Sun S. Cheah C. S. L. (2016). Multitrait–multimethod analysis of the strengths and difficulties questionnaire in young Asian American children. Assessment, 23(5), 603–613. 10.1177/1073191115586459 25979946

